# Testing an audit and feedback-based intervention to improve glycemic control after transfer to adult diabetes care: protocol for a quasi-experimental pre-post design with a control group

**DOI:** 10.1186/s12913-019-4690-0

**Published:** 2019-11-25

**Authors:** Rayzel Shulman, Ian Zenlea, Baiju R. Shah, Cheril Clarson, Jennifer Harrington, Alanna Landry, Zubin Punthakee, Mark R. Palmert, Geetha Mukerji, Peter C. Austin, Janet Parsons, Noah Ivers

**Affiliations:** 1 Division of Endocrinology, The Hospital for Sick Children, Toronto, ON Canada; 2 SickKids Research Institute, Toronto, Canada; 3 ICES, Toronto, Canada; 4 Institute for Health Policy, Management and Evaluation, University of Toronto, Toronto, Ontario Canada; 5 Department of Pediatrics, University of Toronto, Toronto, Canada; 6 Institute for Better Health, Trillium Health Partners, Missisauga, Canada; 7 Department of Medicine, University of Toronto, Toronto, Canada; 8 Department of Medicine, Sunnybrook Health Sciences Centre, Toronto, Canada; 9 Children’s Hospital, London Health Sciences Centre, London, Ontario Canada; 10 Lawson Health Research Institute, London, Ontario Canada; 11 Markham Stouffville Hospital, Markham, Canada; 12 McMaster University, Hamilton, Ontario Canada; 13 Department of Physiology, University of Toronto, Toronto, Ontario Canada; 14 Women’s College Institute for Health System Solutions and Virtual Care, Toronto, Ontario Canada; 15 Applied Health Research Centre, Li Ka Shing Knowledge Institute, St. Michael’s Hospital, Toronto, Ontario Canada; 16 Department of Physical Therapy, University of Toronto, Toronto, Ontario Canada; 17 Department of Family Medicine, Women’s College Hospital, University of Toronto, Toronto, Canada

**Keywords:** Health services research, Audit and feedback, Type 1 diabetes, Transition to adult care

## Abstract

**Background:**

When young adults transfer from pediatric to adult diabetes care they are at risk for deterioration of glycemic control, putting them at an increased risk of developing both acute and chronic complications. Despite increased awareness of these risks, there are gaps in care delivery during this vulnerable time and variability in the implementation of recommended transition practice. Audit and feedback (AF) interventions have a positive but variable effect on implementation of best practices. An expert group identified specific suggestions for optimizing the effectiveness of AF interventions. We aim to test an AF-based intervention incorporating these specific suggestions to improve transition practices and glycemic control in the first year after transfer from pediatric to adult diabetes care.

**Methods:**

This is a pragmatic quasi-experimental study; a series of three cohort studies (pre-implementation, early-implementation, and post-implementation) to compare the baseline adjusted hemoglobin A1c (HbA1c) in the 12 months after the final pediatric visit in five pediatric diabetes centres within the Ontario Pediatric Diabetes Network in Ontario, Canada. The intervention includes three components: 1) centre-level feedback reports compiling data from chart abstraction, linked provincial administrative datasets, and patient-reported experience measures; 2) webinars for facilitated conversations/coaching about the feedback; and 3) online repository of curated transition resources for providers. The primary outcome will be analyzed using a multivariable linear regression model. We will conduct a qualitative process evaluation to understand intervention fidelity and to provide insight into the mechanisms of action of our results.

**Discussion:**

There is a need to develop an innovative system-level approach to improve outcomes and the quality of care for young adults with type 1 diabetes during the vulnerable time when they transfer to adult care. Our research team, a collaboration of health services, implementation science, and quality improvement researchers, are designing, implementing, and evaluating an AF-based intervention using recommendations about how to optimize effectiveness. This knowledge will be generalizable to other care networks that aim to deliver uniformly high-quality care in diverse care settings.

**Trial registration:**

ClinicalTrials.gov NCT03781973. Registered 13 December 2018. Date of enrolment of the first participant to the trial: June 1, 2019.

## Background

For individuals with type 1 diabetes, optimizing glycemic control is critical for preventing chronic diabetes complications [1, 2]. Glycemic control deteriorates in adolescence and remains suboptimal throughout the early to mid-20s [3]. This is particularly concerning because glycemic control early in life has a lasting effect on the future risk of developing long-term diabetes complications [1, 2]. Further, during the transfer to adult care, emerging adults are at high risk of dropping out of medical care [4–8]. This may be due to a number of recognized barriers for emerging adults, including feeling unprepared for transition, difficulties scheduling appointments with and making a connection with the new adult team, and lack of perceived value in the clinic visit [9–11]. We have shown that there is variability in practice regarding transition to adult diabetes care and gaps in transition care across Ontario pediatric diabetes centres [12]. Improved transition preparation, care coordination, and communication between pediatric and adult providers are needed. However, there is limited evidence to support any particular model of transition care for adolescents with chronic conditions in general and diabetes in particular [13–21].

There is an evidence-based, standardized approach with sample tools for providers called ‘Got Transition’ that recommends six core elements of high-quality transition care [22, 23]. The Got Transition/Center for Health Care Transition Improvement is a United States-based cooperative agreement between the Maternal and Child Health Bureau and The National Alliance to Advance Adolescent Health. Implementation of this framework in a pediatric and adult subspecialty diabetes practice improved transition process measures and was associated with stable glycemic control [24]. Audit and feedback (AF) is a quality improvement (QI) approach that involves measuring the quality of care, comparing these results to standards or peer performance, and providing feedback to providers [25]. AF interventions have a positive but variable effect on performance [26, 27]. AF approaches using data from national diabetes registries together with benchmarking against quality indicators to improve pediatric diabetes outcomes have been effective [28–30]. An expert group identified specific suggestions for optimizing the effectiveness of practice feedback interventions through expert interviews; systematic reviews; and experience with providing, evaluating, and receiving practice feedback [31]. However, there is little evidence regarding the effectiveness of AF that include key ‘active ingredients’ and of the underlying mechanisms to support the optimal methods for implementation of such interventions [31].

Ivers et al. have suggested that, in order to advance the field of AF, approaches to optimize and test interventions should be done in collaboration with implementation scientists and health systems or organizations in the context of ‘implementation science laboratories’ [26, 32]. In this article, we describe a study designed by a collaboration of researchers in implementation science, quality improvement, health services, and pediatric diabetes care to test an AF-based intervention that incorporates features identified as key ‘active ingredients’ [31].

In this study we aim to test the effectiveness of an AF-based intervention to improve diabetes control in the first year after youth transfer from pediatric to adult diabetes care. We will also conduct a process evaluation to assess the intervention fidelity and to understand underlying mechanisms to explain our results [33]. We hypothesize that young adults exposed to the AF-based intervention will have better glycemic control 12 months after their final pediatric visit compared to those not exposed.

## Methods

We will conduct a pragmatic quasi-experimental study to assess the impact of our AF-based intervention. We adhered to the SPIRIT (Standard Protocol Items: Recommendations for Interventional Trials) guideline for a clinical trial protocol [34]. This will comprise a series of three cohort studies (pre-implementation, early-implementation, and post-implementation) to compare the baseline adjusted hemoglobin A1c (HbA1c) 12 months after the final pediatric visit [35]. Ethics approval for this protocol was granted by the Clinical Trials Ontario Streamlined Research Ethics Review System and the Trillium Health Partners Research Ethics Board. We will conduct a qualitative process evaluation to explore potential underlying mechanisms of our results [36, 37]. The trial is registered on ClinicalTrials.gov (NLM trial registration number: NCT03781973).

### Setting

In Ontario approximately 7000 children and adolescents live with type 1 diabetes and are cared for by one of the 35 centers in the Ontario Pediatric Diabetes Network (PDN) [38]. In Ontario, youth typically transfer to adult care between age 17 and 19 years [39]. This study will be conducted at five pediatric diabetes centres (three tertiary centres and 2 large community centres) within the Ontario PDN; The Hospital for Sick Children; Trillium Health Partners; Markham Stouffville Hospital; Children’s Hospital, London Health Sciences Centre; and McMaster University Medical Centre. Usual transition care, which is not standardized across sites, will continue during the study period.

### Intervention design

The AF-based intervention targets healthcare providers who are pediatric diabetes team members at the participating study sites and was designed using the following four categories of key recommendations for designers of practice feedback and implementation strategies: 1) the nature of the desired action; 2) nature of the data available for feedback; 3) feedback display; 4) and delivering the feedback intervention [31].

#### 1) The nature of the desired feedback:

Feedback that supports actions that are consistent with established goals and priorities is more likely to be effective [31]. Therefore, we will recommend actions that are consistent with established goals and priorities by introducing ‘Got Transition’, Six Core Elements of Health Care Transition 2.0, a US- funded initiative, that developed a set of six core elements of quality transition care; 1) Transition policy; 2) Transition tracking and monitoring; 3) Transition readiness; 4) Transition planning; 5) Transfer of care; and 6) Transfer completion [40, 41]. These resources will inform providers about best transition practices and provide useful tools to facilitate local quality improvement initiatives [42, 43]. The first report will contain baseline data about transition performance of each site.

#### 2) & 3) The nature of the data available for feedback and feedback display:

Brehaut et al. suggest providing multiple instances of feedback and to closely link the visual display and summary message [31]. As such, we will provide multiple instances of site-specific aggregate feedback; reports every six months during the intervention as described below.

##### Feedback reports

The reports will be populated with data collected from three sources described in detail in the data sources section below: 1) medical record data abstracted from patient charts; 2) provincial population-based administrative databases held at ICES (formerly the Institute of Clinical and Evaluative Sciences); and 3) patient-reported transition experience and outcome measures. The data in the reports will be organized according to the ‘Got Transition’ six core elements [23]. We will display the feedback in the reports with summary messages at the top of each section of data. A template of the centre performance feedback report is available in Additional file 1.

#### 4) Delivering the feedback intervention

##### Webinars

To complement the feedback reports, the study team will host a series of webinars for provider participants every three months to give providers an opportunity to reflect on the feedback report and to have facilitated conversations/coaching about the feedback. There will be a total of eight webinars, each scheduled to last 45 min. During the first webinar, the study team will guide participants about how to interpret the report and address the limitations of the feedback data. Sites will also be able to compare their own performance to that of all other centres (anonymously). Each subsequent webinar will be co-hosted by the study team and one study site. Each site will share an example of a QI initiative that they are executing and describe their success and challenges. The study team will post a one-page summary of the main messages discussed at each webinar. Participation in webinars and frequency of accessing the summaries will be monitored and used as part of the process evaluation.

##### Online resources

As a supplement to the “Got Transition” Six Core Elements, we will post additional transition resources that currently exist in the public domain in a dedicated space for study materials within a provincial QI online forum, call Quorum [44]. The resources were selected from those recommended in the Ontario PDN Transition to Adult Care Working Group Recommendations Report [45]. These resources will be available to health care provider participants to use as part of their QI initiative if they choose. We will track the frequency that the resources on the study website are accessed as part of our process evaluation.

### Participants

Health care providers: All health care professionals (i.e., physicians (including endocrinology residents), nurses, dieticians, social workers, and psychologists), who are members of the diabetes healthcare team at participating sites, will be invited to participate in the intervention. Health care providers will be invited to use the feedback reports, participate in the webinars, and access the online resources to facilitate transition care related QI activities within their diabetes healthcare teams. Each team will identify a local “transition champion” to lead local team discussions aimed at identifying areas in need of improvement based on the feedback report and to develop and execute quality improvement projects designed to address the gap(s).

Patients: Three separate patient cohorts will be constructed: (i) those whose last pediatric visit was in the year before the intervention (pre-implementation); (ii) those whose last pediatric visit was in the year immediately after the start of the intervention (early-implementation); (iii) those whose last pediatric visit was in the second year after the intervention (post-implementation). We estimate that our sample size will be approximately 225 individuals per cohort, with individual sites contributing between 25 and 70 individuals to each cohort.

We will invite all participants in the early and post-intervention study cohorts to complete the patient experience surveys. Each site will pre-identify eligible participants who are due to come to clinic for their final pediatric visit. We will mail or email (depending on site-specific clinic practice) an information letter, signed by their own diabetes team, describing our study to all eligible participants. The letter will contain an opt-out contact email and phone number if they do not want to be contacted about participating. The letter will explain our research project, what data we plan to collect and how we will use it. At their final pediatric visit, a research coordinator will meet the participant and invite them to complete the baseline survey and confirm a mailing and email addresses for the 12 month follow-up survey. We will offer an incentive gift card to participants for each survey they complete as a strategy for achieving adequate participant enrolment.

All youth living with type 1 diabetes followed at participating sites will enter the study at the time of their final pediatric clinic visit and will be followed for a minimum of 12 months up to the end of the study period. The final pediatric visit will be the visit at which a referral to an adult diabetes physician is made and is planned to be the final pediatric visit. We will exclude individuals with non-type 1 diabetes, those who plan to move out of Ontario within 12 months after their final pediatric visit, and those who do not have the capacity to consent for themselves.

### Data collection

Data for use in the feedback reports will be derived from medical charts, routinely collected provincial administrative data linked using unique encoded identifiers at ICES, and patient-reported transition experience measures (for those cohorts ii and iii). The provincial health insurance number and participants’ study IDs will be securely transferred to ICES and linked to ICES databases. The study investigators will be permitted to access de-sensitized information only for analysis (i.e., any information that can directly identify a person such as the provincial health insurance number or name will be removed or replaced with a code that is not known to the study investigators).

#### Chart data

Data abstracted from the chart will include, Ontario Health Insurance Plan (OHIP) number (to link to administrative datasets at ICES), date of diabetes diagnosis, last HbA1c available while in pediatric care (within 12 months before the final pediatric visit), method of HbA1c collection (lab vs. point of care testing (POCT)), HbA1c laboratory assay or POCT testing platform, the date of the final pediatric visit, and the name of the adult diabetes clinic to which the patient is referred.

#### ICES data

We will extract data from the following ICES databases: 1) Ontario Lab Information System (OLIS)(to measure HbA1c while in adult care); 2) the Hospital Discharge Abstract Database (information on discharges from acute care facilities); 3) the Ontario Health Insurance Plan Database (physician billing claims); 4) the National Ambulatory Care Reporting System (information on emergency department visits); 5) the Registered Persons Database (demographics and vital statistics including outmigration of all legal residents in Ontario); 6) the Ontario Registrar General-Death for cause of death; 7) the Assistive Devices Program (ADP) to identify claims for insulin pumps and supplies; 8) the 2016 Canadian Census to assign neighbourhood material deprivation quintiles to Ontario residents; 9) The Ontario Mental Health Reporting System (data on patients in inpatient mental health beds); and 10) ICES physician database (identifies physician speciality).

#### Patient experience measures

We will conduct a patient transition experience survey at the final pediatric visit (baseline) and 12 months later for the early and post-implementation cohorts only. We modified the survey used in the T1D Exchange Transition Experiences Study which includes the following domains: 1) Current Diabetes Care; 2) Pediatric Diabetes Care; 3) Transition from Pediatric to Adult Care; 4) Current Diabetes Self-Care and Support; 5) Social Networking; and 6) Demographics (Additional file 2) [7]. The results of the survey will be included in the performance feedback reports to centres. These will not be used as an outcome measure since these will not be measured in the pre-implementation cohort.

### Outcomes

The outcomes and covariates, period of ascertainment, and data sources are summarized in Table 1. The primary outcome is HbA1c (the last HbA1c value available during the 12 months after the final pediatric visit), ascertained from OLIS. We will adjust for the baseline HbA1c at the time of the final pediatric visit in the regression model, which will be ascertained from the medical record, because not all pediatric hospital laboratory data are available in OLIS. Secondary outcomes will include the occurrence of at least one diabetes-related admission or emergency department visit, or all-cause death in the 12 months after the final pediatric visit. We will use ICD-10-CA codes for diabetes-related preventable hospitalizations to identify diabetes-related admissions [46, 47]. We will measure all-cause death, as not all sources of mortality data include cause of death. We will also measure the time from the final pediatric visit (extracted from the medical record) to the first adult diabetes visit, identified using physician service claims and defined as the first diabetes office visit by an adult endocrinologist or internist after the final pediatric visit.

**Table 1:** Outcomes and covariates, period of ascertainment, and data sources

Variable	Period of ascertainment	Data sources
Primary outcome		
Most recent HbA1c 12 months after the final pediatric visit	12 months after the final pediatric visit	Ontario Lab Information System (OLIS)
Secondary outcomes		
At least one diabetes-related admission	12 months after the final pediatric visit	Hospital Discharge Abstract Database (DAD)
At least one diabetes-related emergency department visit		National Ambulatory Care Reporting System (NACRS)
Death		Registered Persons Database (RPDB) and the Ontario Registrar General-Death (ORGD)
Gap in time (months) from the final pediatric visit to first adult diabetes visit		Medical record (date of final pediatric visit) and OHIP and ICES physician database (date of the first adult diabetes visit)
Covariates		
Sex	At the time of the final pediatric visit	Medical record
Duration of diabetes		Medical record (date of diabetes diagnosis)
Rurality		Postal code ascertained from the medical record and the Rurality Index for Ontario
Socioeconomic status (deprivation quintile)		Postal codes ascertained from the medical record and 2006 Canadian Census to assign neighbourhood material deprivation quintiles
Most recent HbA1c at the time of the final pediatric visit	24 months prior to the final pediatric visit	Medical record
Any diabetes-related admission		Hospital Discharge Abstract Database (DAD)
Any diabetes-related emergency department visit		National Ambulatory Care Reporting System (NACRS)
Any mental health visit		Ontario Mental Health Reporting System and OHIP

#### Exposures

The main exposure is the cohort variable: (i) those youth whose last pediatric visit was in the year before the intervention (pre-implementation); (ii) those whose last pediatric visit was in the year immediately after the start of the intervention (early-implementation); and (iii) those whose last pediatric visit was in the second year after the intervention (post-implementation).

#### Other variables

We will estimate socioeconomic status using household income, assigned ecologically at the neighbourhood level using postal codes, divided into quintiles [48]. Other covariates are sex and duration of diabetes on the date of the final pediatric visit. We will use the Rurality Index for Ontario, based on community characteristics including access to health services, to categorize rurality [49]. We will measure the presence of any prior acute diabetes complications (diabetes-related admissions and emergency department visits) and any prior mental health visit (either hospital admission or physician visit for a mental health problem) within 24 months prior to the final pediatric visit as baseline covariates. A list of mental health diagnostic codes used to identify outpatient physician visits for a mental health problem are available in Additional file 3.

### Analysis

We will describe the baseline characteristics and the occurrence of any missing data for each cohort using descriptive statistics. We will test the effectiveness of the intervention using a multivariable linear regression model that regresses HbA1c (the last HbA1c value available during the 12 months after the final pediatric visit) on the main exposure variable (the main exposure variable is the cohort variable) using data from all three cohorts pooled together. The model will also adjust for the following variables: last recorded value of HbA1c at the final pediatric visit, pediatric diabetes centre, and the covariates listed above. Our sample size of 675 individuals in 3 cohorts provides us with 92% power to detect a difference in means between the three cohorts, based on analysis of covariance, in which we adjust for baseline HbA1c and the other variables described above. This assumes that the mean HbA1c in the control and early-intervention cohorts is 8.82% and that it is 8.32% in the late-intervention cohort, with a common standard deviation of 1.75. This is based on the observation that the mean HbA1c and standard deviation for all individuals aged 18–24 years with diabetes diagnosed before age 15 years in Ontario from 2011 to 2014 was 8.82% ±1.75 (unpublished). We will also include an interaction term between the cohort variable and diabetes centre to determine if the effect of the intervention is different depending on the pediatric centre. We will also report sex-stratified outcomes.

We will assess the effect of the intervention on the occurrence of any acute diabetes complication or death using a logistic regression model. We will assess the effect of the intervention on the time to first adult diabetes visit using a Cox proportional hazards model. For both models, the main exposure variable will be the cohort (pre-implementation vs. early-implementation vs. post-implementation). We will also adjust for pediatric diabetes centre (5 levels) and the covariates listed above.

### Process evaluation

The quantitative data collected to assess the outcomes of the trial are important to determining *if* the intervention works. However qualitative approaches are particularly useful for understanding *how* and *why* a given intervention works or not [36]. We will conduct a qualitative process evaluation to understand whether the intervention was received as intended (i.e., intervention fidelity), the mechanisms of action, and the contextual features associated with engagement with the intervention (i.e., conditions and factors that might affect trial results at each of the study sites, as well as macro level health system factors) [50]. Data sources will include focus groups with diabetes team members, conducted following the receipt of first feedback and at end of the trial [38]. There will be a set of two initial focus groups conducted virtually by videoconference with the site champions from each of the participating sites [40]. The rationale for the initial set of focus groups is to understand the trial as it is unfolding (and to explore how the feedback is being received). This will allow us to address any challenges to implementation, with a view to optimizing recruitment/participation in the final round of focus groups, as well as informing data collection procedures at end of trial (e.g., refinement of final focus group guide for the process evaluation). At the end of the trial we will conduct one focus group with all available diabetes team members at each of the participating sites. For all focus groups, we will use a semi-structured interview guide informed by the consolidated framework for advancing implementation science to capture diabetes team members’ experiences of participating in the QI intervention [41]. For the final round of focus groups, we will aim to include between 6 and 8 participants per group [42]. We will audio record and transcribe the focus groups. Focus groups will all be conducted by experienced qualitative researchers. During focus groups two researchers will be present – one to facilitate the group and one to take detailed field notes of the discussion (e.g., group dynamics, non-verbal communication, etc.). We will use a qualitative descriptive approach to analyze the focus group transcripts [43]. Constant comparison will be conducted to look for differences and similarities within and across groups and between data sources [51].

## Discussion

There is a need to develop an innovative system-level approach to improve outcomes and the quality of care for youth during the vulnerable time while they transition from pediatric to adult diabetes care. We are taking advantage of an existing network of pediatric diabetes programs that is committed to improving the quality of care, an accepted general transition care practice guideline, and population-level provincial administrative datasets to design, implement, and evaluate an AF-based intervention using recommendations about how to optimize effectiveness [32]. Our findings will serve as a foundation on which to build future trials embedded within existing QI programs to optimize the effectiveness of AF and transition practice.

Improving outcomes of and the quality of diabetes transition care is a key priority for the Ontario PDN and other national and international diabetes organizations [45, 52, 53]. Unlike existing models of transition care that are limited by their cost, scalability, and generalizability, our approach can be expanded in scale and modified to function in local contexts and used in conjunction with existing diabetes registries and administrative datasets. Our intervention can be translated immediately into clinical practice and will have a direct impact on how services are delivered to youth with diabetes.

Further, our embedded process evaluation aims to advance knowledge about the mechanisms underlying the effectiveness of specific components of AF and to assess the variable impact of different components [31]. Results of the process evaluation will inform future work to refine the optimal design, delivery, and additional features of our AF-based intervention. We anticipate using this knowledge to address some of the limitations of our study. One such limitation is the risk of poor provider engagement. To mitigate this risk, study teams have agreed to participate in our webinars during their regularly scheduled team meeting times in order to minimize the burden on providers. Further, we hope that the discussion about the feedback reports, coaching, and availability of online transition resources will incentivize provider participation in the webinars and ignite enthusiasm for local QI initiatives. In order to maximize patient enrollment, we are offering monetary incentives for completing the baseline and 12 month follow-up patient surveys.

Further, we are unable to identify diabetes visits to non-physician providers from our administrative data. Therefore, we may over-estimate the time from the last pediatric visit to the first adult diabetes visit if the first visit was to a diabetes nurse or dietician educator. Finally, we recognize that delivery of adult diabetes care is an important contributing factor to transition outcomes. It is not feasible to include adult diabetes centres in this study because only small numbers of patients are transferred to each of the many adult diabetes program each year; however, we have three adult diabetes providers on our research team to provide an adult provider perspective and we will provide a study summary report to local adult diabetes programs.

We are using this study as a foray to develop an “implementation laboratory” within the Ontario PDN [32, 54]. We envision an ongoing collaboration between the PDN and our research team to embed implementation research within the fabric of its quality improvement initiatives. The PDN is committed to QI and is currently undertaking evaluative processes to inform its QI strategies. The PDN is an ideal setting for an implementation laboratory because there are health system characteristics common to all PDN sites, yet each of the 35 sites resides in a unique context in which there is an opportunity to explore how local factors modify the effect of an intervention [32]. Given that members of our research team are also members of the PDN Quality and Clinical Standards Committee, we can work to ensure that the goals and objectives of both our research team and the PDN are aligned and met.

Looking ahead, Health Quality Ontario is currently developing quality standards for care for people living with type 1 diabetes expected to be available in spring/summer 2020. These quality statements accompanied by their structure, process and/or outcome indicators, could be incorporated into implementation strategies and tested in this proposed implementation laboratory. Our goal is to generate evidence that is generalizable to other care networks that aim to deliver uniformly high quality care in diverse care settings.

## Supplementary information

**Additional file 1.** Feedback Report Template.

**Additional file 2.** Patient experience surveys (baseline and 12 month follow-up).

**Additional file 3.** Mental Health Diagnostic Codes.

## Data Availability

Not applicable.

## Abbreviations

AF: Audit and feedback
HbA1c: Hemoglobin A1c
OLIS: Ontario Lab Information System
PDN: Pediatric Diabetes Network
QI: Quality improvement
