# Supplementary material for: Testing an audit and feedback-based intervention to improve glycemic control after transfer to adult diabetes care: protocol for a quasi-experimental pre-post design with a control group
Source: BMC Health Serv Res. 2019 Nov 25;19:885. doi: 10.1186/s12913-019-4690-0 (PMC6878686; doi:10.1186/s12913-019-4690-0)
Supplement: Supplementary file 1 — Additional file 1. Feedback Report Template. [file 12913_2019_4690_MOESM1_ESM.docx]

Bridging the gap to optimize care and outcomes for youth with diabetes between pediatric and adult diabetes care

Feedback Report Template

Centre name: _________________

Distribution date: _______ (Includes data from ___ to ____)

This report contains information about patients followed at your centre who had their final pediatric visit between ____ and ________ compared to patients followed at the other 4 study sites.

Data used to create this report come from:

- Health records at your site
- Provincial administrative databases held at the Institute for Clinical and Evaluative Sciences (ICES)
- Patient experience surveys

Contents of this report:

1. Your data, presented according to the Six Core Elements of Healthcare Transition from the Got Transition framework (see explanation in the next section). At the beginning of each section we will include your site’s self-assessment about the extent to which the Six Core Elements of Health Care Transition 2.0 are being incorporated into clinical processes.
2. How to interpret these data?
3. How to use these data?

Executive summary:

Your centre has much to be proud of with regard to how it cares for children with diabetes. *XX% of patients from your centre felt satisfied with their transition from pediatric to adult care.* In this learning community, you’ll be able to share your areas of strength and learn from others about how to make change in your centre prior to transition that will benefit these patients long after they transition.

The **Six Core Elements of Health Care Transition 2.0** described in this report in grey boxes below are intended for use by pediatric, family medicine, med-peds, and internal medicine practices to assist youth and young adults as they transition to adult-centered care. They are aligned with the AAP/AAFP/ACP Clinical Report on transition^1^.

<https://www.gottransition.org/providers/index.cfm>

1 American Academy of Pediatrics, American Academy of Family Physicians, American College of Physicians. Transitions Clinical Report Authoring Group. Supporting the health care transition from adolescence to adulthood in the medical home. Pediatrics. 2011; 128:182.

| 1. Transition Policy |
| --- |
| - Develop a transition policy/statement with input from youth and families that describes the practice’s approach to transition, including privacy and consent information. - Educate all staff about the practice’s approach to transition, the policy/statement, the Six Core Elements, and distinct roles of the youth, family, and pediatric and adult health care team in the transition process, taking into account cultural preferences. - Post policy and share/discuss with youth and families, beginning at age 12 to 14, and regularly review as part of ongoing care. |

- *Site-specific “Current Assessment of Health Care Transition Activities for Transitioning Youth to Adult Health Care Providers” Transition Policy: levels 1-4*

| 2. Transition Tracking and Monitoring |
| --- |
| - Establish criteria and process for identifying transitioning youth and enter their data into a registry. - Utilize individual flow sheet or registry to track youth’s transition progress with the Six Core Elements. - Incorporate the Six Core Elements into clinical care process, using EHR if possible. |

- *Site-specific “Current Assessment of Health Care Transition Activities for Transitioning Youth to Adult Health Care Providers” Tracking and Monitoring: levels 1-4*

How many individuals do you discharge from your clinic and to which adult diabetes sites are they most frequently referred?

|  | **Your site** | **All other study sites combined** |
| --- | --- | --- |
| Total number of individuals discharged from pediatric care |  |  |
| Number of individuals referred to site: |  |  |
| A |  |  |
| B |  |  |
| C |  |  |

| 3. Transition Readiness AND 4. Transition Planning |
| --- |
| - Conduct regular transition readiness assessments, beginning at age 14, to identify and discuss with youth and parent/caregiver their needs and goals in self-care. - Jointly develop goals and prioritized actions with youth and parent/caregiver, and document regularly in a plan of care. - Develop and regularly update the plan of care, including readiness assessment findings, goals and prioritized actions, medical summary and emergency care plan, and, if needed, a condition fact sheet and legal documents. - Prepare youth and parent/caregiver for adult approach to care at age 18, including legal changes in decision-making and privacy and consent, self-advocacy, and access to information. - Determine level of need for decision-making supports for youth with intellectual challenges and make referrals to legal resources. - Plan with youth/parent/caregiver for optimal timing of transfer. If both primary and subspecialty care are involved, discuss optimal timing for each. - Obtain consent from youth/guardian for release of medical information. - Assist youth in identifying an adult provider and communicate with selected provider about pending transfer of care. - Provide linkages to insurance resources, self-care management information, and culturally appropriate community supports. |

- *Site-specific “Current Assessment of Health Care Transition Activities for Transitioning Youth to Adult Health Care Providers” Transition readiness: levels 1-4*
- *Site-specific “Current Assessment of Health Care Transition Activities for Transitioning Youth to Adult Health Care Providers” Transition Planning: levels 1-4*

Baseline characteristics associated with a high risk of adverse events during early adulthood*

| **At the time of the final pediatric visit** | Your centre | All other study centres |
| --- | --- | --- |
| Most recent HbA1c  mean (range) or median (IQR)  >9.0%, n (%) |  |  |
| Number (%) in the bottom 2 quintiles for material deprivation |  |  |
| **In the 24 months before the final pediatric visit** | | |
| 1 or more diabetes-related admissions |  |  |
| 1 or more diabetes-related emergency department visits that did result in an admission |  |  |
| 1 or more mental health physician visits |  |  |

*For privacy reasons, we cannot reveal the identities of these individuals to you

**Summary**: patients referred to adult care from your centre………..

Baseline patient experience survey data. This survey was sent to XX patients from your centre, at time X, identified by means Y. Response rate was Z.

| **In the 12 months before the planned transfer to adult care individuals report that:** | | | | |
| --- | --- | --- | --- | --- |
|  | Your centre  N= | | All other study centres  N= | |
|  | N(%) reported this occurred | N(%) felt this is important or very important | N(%) reported this occurred | N(%) felt this is important or very important |
| 1. Met pediatric diabetes provider without parent/guardian in the room |  |  |  |  |
| 1. Pediatric diabetes provider recommended a specific adult diabetes clinic or provider |  |  |  |  |
| 1. Received contact information for the new adult diabetes clinic or provider |  |  |  |  |
| 1. Had a visit with a pediatric diabetes provider specifically to talk about transition |  |  |  |  |
| 1. Received written materials about transitioning to adult diabetes care |  |  |  |  |
| 1. Talked with pediatric diabetes provider about independently managing diabetes |  |  |  |  |
| 1. Talked with pediatric diabetes provider about screening tests for diabetes complications |  |  |  |  |
| 1. Talked with my pediatric diabetes provider about reproductive health (eg. Pregnancy planning for women and sexual function for men. |  |  |  |  |
| 1. Talked with my pediatric diabetes provider about alcohol with diabetes |  |  |  |  |
| 1. Met with new adult diabetes provider before leaving pediatric diabetes provider |  |  |  |  |
| 1. Talked about how to obtain and pay for diabetes supplies. |  |  |  |  |
| **At the time of the final pediatric visit:** | | | | |
|  | Your centre n= | | All other sites: n= | |
|  | N (%) | | N (%) | |
| Individuals reported feeling either mostly or completely prepared to leave their pediatric diabetes providers |  | |  | |

**Summary:** patients referred to adult care from your centre…..

| 5. Transfer of care AND 6. Transfer completion |
| --- |
| - Confirm date of first adult provider appointment. - Transfer young adult when his/her condition is stable. - Complete transfer package, including final transition readiness assessment, plan of care with transition goals and pending actions, medical summary and emergency care plan, and, if needed, legal documents, condition fact sheet, and additional provider records. - Prepare letter with transfer package, send to adult practice, and confirm adult practice’s receipt of transfer package. - Confirm with adult provider the pediatric provider’s responsibility for care until young adult is seen in adult setting. - Contact young adult and parent/caregiver 3 to 6 months after last pediatric visit to confirm transfer of responsibilities to adult practice and elicit feedback on experience with transition process. - Communicate with adult practice confirming completion of transfer and offer consultation assistance, as needed. - Build ongoing and collaborative partnerships with adult primary and specialty care providers. |

Data about delays in transition to adult care from Institute for Clinical and Evaluative Sciences (ICES) administrative databases and patient experience survey data as of **date X**, considering patients who transitioned between **date y and date z**

|  | **Your site** | **All other study sites combined** |
| --- | --- | --- |
| Time from last pediatric visit to first adult **physician** visit, n (%) | <4 months:  4-6 months:  7-9 months:  10-12 months:  No adult physician visit: | <4 months:  4-6 months:  7-9 months:  10-12 months:  No adult physician visit: |
| **YOUR SITE ONLY:** | | |
| Name of adult diabetes clinic and the number of patients referred to this clinic during the reporting period | | Time from the last pediatric visit to first adult physician visit (n (%)) |
| Clinic A: n | | <4 months:  4-6 months:  7-9 months:  10-12 months:  No adult physician visit: |
| Clinic B: n | | <4 months:  4-6 months:  7-9 months:  10-12 months:  No adult physician visit: |
| Individual physician not affiliated with a diabetes clinic: n | | <4 months:  4-6 months:  7-9 months:  10-12 months:  No adult physician visit: |

**Summary**: Patients referred to an adult diabetes physician from your centre are seen on average x months after their final pediatric visit.

| **Diabetes outcomes in the 12 months after the final pediatric visit** | | |
| --- | --- | --- |
|  | Your centre  N= | All other study centres N= |
| Most recent HbA1c  mean (range) or median (IQR)  >9.0%, n (%) |  |  |
| 1 or more diabetes-related admissions |  |  |
| 1 or more diabetes-related emergency department visits that did result in an admission |  |  |
| 1 or more mental health physician visits |  |  |

| **Patient experience survey data 12 months after the final pediatric visit** | | |
| --- | --- | --- |
|  | Your centre  N= | All other study centres  N= |
|  | N(%) felt this was a moderate or major problem | N(%) felt this was a moderate or major problem |
| The following is a list of problems that individuals living with diabetes have said made it hard for them to get established with an adult diabetes provider after leaving their  pediatric diabetes provider: |  |  |
| a. I didn't have a name for a new adult provider |  |  |
| b. I didn't know how to contact the new adult provider |  |  |
| c. I couldn't get an appointment with the new adult provider |  |  |
| d. I forgot to make an appointment or had other priorities |  |  |
| e. I felt upset about leaving my pediatric diabetes providers |  |  |
|  | Your centre  N= | All other sites:  N= |
| Individuals reported either mostly or completely satisfied with how their transition to adult diabetes care went | N (%) | N (%) |

How to interpret these data?

You might have questions about the validity of the data. Although we are confident that the data we used to create these reports are comprehensive and reliable, there are some issues to be aware of:

- It is possible that there are missing items or incorrect information in the patient chart, however, we collected all available data. There may be additional information about your patients that might be useful, but were not available. For example visits to non-physician health care professionals are not available from ICES, however, we did collect this information in our patient experience survey.
- The ICES databases contain all information about physician visits and hospital admissions in Ontario. If a patient received care outside of Ontario, this information is not included.
- The data we collected from patients about their transition experience might not be representative of the entire patient population.

You might wonder if the patients are your centre are similar to the patients at other study centres. You might also wonder if YOUR patients are different than the patients seen by at your centre overall:

- - In the table in the section about Transition Readiness and Transition Planning (PAGE X) we include some characteristics of patients that are known to be associated with adverse event around transition to adult care. You can see if there are differences in these important characteristics between your patients and those at other centres that might explain differences in outcomes.
  - There may be other differences in characteristics such as level of parental education that we could not measure.
  - If you think there might be differences in your patients or the patients at your site compared to other sites, we encourage you to consider this in your approach to QI at your site.

How to use these data?

You might feel uncertain if you can use these data to help future patients who will transition from your centre, but there are steps you can take that are likely to make a difference:

1. Review the report with your team: QI is a team sport!
2. Discuss how your clinic compares to other study sites and how it compares to your team’s goals
3. Identify an area to target for improvement and develop a specific aim statement
4. Start your QI journey, working together with us to improve transition care for all our patients

Please join us for Session 2 of the Transition to Adult Care Learning Community

Title, session objectives, Date and Time and link to be entered here

ADD LINK TO ONLINE TRANSITION RESOURCES TO BE ENTERED HERE

If you have questions about this report please contact:

Dr. Rayzel Shulman

[Rayzel.shulman@sickkids.ca](mailto:Rayzel.shulman@sickkids.ca)

Ph. 416-813-6218
