# Supplementary material for: Testing an audit and feedback-based intervention to improve glycemic control after transfer to adult diabetes care: protocol for a quasi-experimental pre-post design with a control group
Source: BMC Health Serv Res. 2019 Nov 25;19:885. doi: 10.1186/s12913-019-4690-0 (PMC6878686; doi:10.1186/s12913-019-4690-0)
Supplement: Supplementary file 2 — Additional file 2. Patient experience surveys (baseline and 12 month follow-up). [file 12913_2019_4690_MOESM2_ESM.pdf]

Baseline Survey-Bridging the Gap to Optimize Care and Outcomes for Youth with Diabetes between Pediatric and Adult Diabetes Care

We are conducting this research study to find out about gaps in services and other issues that young adults with type 1 diabetes face during their transition to adult care. We hope to learn how we can give the best possible care for young adults with type 1 diabetes as they move to adult care.

The questions in this survey from your last pediatric clinic visit will ask about your current diabetes self-care and support and your planned health care transition. There are 26 questions and it will take about 15 minutes to complete. By completing the survey, you consent to participate as outlined in the participant letter you received.

You will receive a $10.00 gift card for completing this baseline survey, $10.00 gift card if you complete the 12 month follow-up survey or $25.00 gift card if you complete both the baseline and 12 month follow-up survey.

A member of the research team at your centre will have given you a 'Study ID' number. Please use this number to answer the first question on the survey.

Thank you!

Study ID

____
(you received this from the study team)

Today's date

____

SECTION 1: YOUR CURRENT DIABETES SELF-CARE AND SUPPORT

1. How old are you today?

- ◯ 15
- ◯ 16
- ◯ 17
- ◯ 18
- ◯ 19
- ◯ 20
- ◯ 21

---

2. To what extent are your parents/guardians currently involved in your diabetes care?

- ◯ Not at all involved
- ◯ Somewhat involved
- ◯ Moderately involved
- ◯ Very involved

---

3. How involved would you like your parents/guardians to be in your diabetes care?

- ◯ Not at all involved
- ◯ Somewhat involved
- ◯ Moderately involved
- ◯ Very involved

4. Do you currently use any of the following technology (check all that apply):

- ☐ Insulin pump
- ☐ Continuous glucose monitoring system (eg Dexcom)
- ☐ Flash glucose monitoring system (eg Freestyle Libre)
- ☐ Other

Other technology: ______________________

5. In general, how often do you feel stressed because of your diabetes?

- ◯ Never
- ◯ Rarely
- ◯ Sometimes
- ◯ Often
- ◯ Always

6. How often does your diabetes prevent you from doing the following:

	< once per month	1-2 times per month	3-4 times per month	> 4 times per month
Exercise	◯	◯	◯	◯
Sleep	◯	◯	◯	◯
Attend school	◯	◯	◯	◯
Attend work	◯	◯	◯	◯
Participate in social activities	◯	◯	◯	◯

7. In the last year, have you experienced a major change in your life situation that caused you to feel "stressed" or have a physical, mental, or emotional response for an extended period of time?

- ◯ Yes
- ◯ No
- ◯ Don't know

8. If YES: In the past year, which of the following events have you experienced? Please check all that apply.

- ☐ Serious illness or injury in me
- ☐ Serious illness or injury in a family member
- ☐ Hospitalization of a family member
- ☐ Death of a family member
- ☐ Death of a friend
- ☐ Separation or divorce of parents
- ☐ Split up with boyfriend/girlfriend
- ☐ Moved to a new home
- ☐ Started attending a new school
- ☐ Started working at a new job
- ☐ Problems at work or school
- ☐ Lost my job
- ☐ Family member lost his/her job
- ☐ Financial problems in the family
- ☐ Legal problems in the family
- ☐ Other event (please specify):

Other event: ______________________

SECTION 2: YOUR PLANNED HEALTH CARE TRANSITION

The following questions ask about your plans for transition from pediatric to adult care, the time in your life after you stop seeing pediatric health care providers for your diabetes and start seeing an adult healthcare provider for your diabetes.

9. What are the reasons you plan to leave your pediatric diabetes providers? Check all that apply.

- ☐ My pediatric provider suggested that I transition to adult care
- ☐ My parent/guardian suggested that I transition to adult care
- ☐ I feel like I am too old to keep coming to a pediatric diabetes provider
- ☐ I don't like my pediatric diabetes provider
- ☐ I am moving
- ☐ I am going away to college
- ☐ I am pregnant
- ☐ It was mandatory that I transition to adult care
- ☐ Other reason (please specify):

Other reason: __________________________________

10. On the below list, please check the single most important reason you have for leaving your pediatric diabetes providers.

- ◯ My pediatric provider suggested that I transition to adult care
- ◯ My parent/guardian suggested that I transition to adult care
- ◯ I feel like I was too old to keep coming to a pediatric diabetes provider
- ◯ I don't like my pediatric diabetes provider
- ◯ I am moving
- ◯ I am going away to college
- ◯ I am pregnant
- ◯ It is mandatory for me to transition to adult care
- ◯ Other reason (please specify):

Other single most important reason: __________________________________

11. Did the following things happen with any member of your pediatric diabetes team (eg doctor, nurse, dietician, social worker etc) in the past 12 months before you plan to transition from pediatric to adult care? Select one response for each item:

	Yes	No	Don`t know
a. My pediatric diabetes provider and I met without my parent/guardian in the room.	○	○	○
b. My pediatric diabetes provider recommended a specific adult diabetes clinic or provider.	○	○	○
c. My diabetes provider gave me contact information for the new adult diabetes clinic or provider.	○	○	○
d. I had a visit with a pediatric diabetes provider specifically to talk about transitioning to adult diabetes care.	○	○	○
e. My pediatric diabetes provider gave me written or online materials about transitioning to adult diabetes care.	○	○	○
f. My pediatric diabetes provider and I talked about me managing my own diabetes (for example, checking my blood sugar levels, taking my insulin, reviewing blood sugar data, adjusting my insulin.	○	○	○
g. My pediatric diabetes provider and I talked about the diabetes screening tests I need to have (for example, eye exam, blood and kidney tests).	○	○	○
h. My pediatric diabetes provider and I talked about reproductive and sexual health (for example, birth control and pregnancy planning for women and sexual function for men).	○	○	○
i. My pediatric diabetes provider and I talked about drinking alcohol and using recreational drugs (eg. marijuana) with diabetes.	○	○	○

j. I met my new adult diabetes provider before I left my pediatric diabetes provider.	◯	◯	◯
k. My pediatric diabetes provider and I talked about how I would get and pay for my diabetes supplies.	◯	◯	◯

---

12. To what extent do you feel prepared or unprepared to leave your pediatric diabetes providers?

- ◯ Completely unprepared
- ◯ Mostly unprepared
- ◯ Neutral
- ◯ Mostly prepared
- ◯ Completely prepared

13. How important or not important do you think each of the following is in helping people with diabetes transition to adult care?

	Not at all important	A little important	Important	Very Important
a. Having visits with pediatric diabetes provider without a parent/guardian in the room.	○	○	○	○
b. Having a pediatric diabetes provider recommend an adult diabetes clinic or provider.	○	○	○	○
c. Having a pediatric diabetes provider give contact information for the adult clinic or provider.	○	○	○	○
d. Having a visit with a pediatric diabetes provider to talk about transitioning to adult care.	○	○	○	○
e. Reading written or online materials about transitioning to adult diabetes care.	○	○	○	○
f. Talking with a pediatric diabetes provider about managing diabetes (for example, checking blood sugar levels, taking insulin, reviewing blood sugar data, adjusting insulin).	○	○	○	○
g. Learning about the screening tests people with diabetes need to get.	○	○	○	○
h. Talking with a pediatric diabetes provider about reproductive and sexual health (for example birth control and pregnancy planning for women and sexual function for men).	○	○	○	○
i. Talking with a pediatric provider about drinking alcohol and using recreational drugs (eg. marijuana) with diabetes.	○	○	○	○
j. Meeting the adult provider before transitioning to the adult clinic.	○	○	○	○
k. Talking with a pediatric provider about how to get and pay for diabetes supplies.	○	○	○	○

14. What else do you think would be helpful in the transition to adult diabetes care? ______________________________

SECTION 3: SOCIAL NETWORKING

15. Do you currently use any general online social networks (e.g., social networks like Facebook that do not focus on diabetes)?

◯ Yes
◯ No

16. Which general social networks do you use most often? CHECK UP TO 3 CHOICES.

☐ Facebook
☐ Flickr
☐ Google+
☐ Instagram
☐ LinkedIn
☐ Pinterest
☐ Snapchat
☐ Tinder
☐ Tumblr
☐ Twitter
☐ None
☐ Other (please specify)

Other general social network: __________________________________

17. Do you currently use any social networking sites for diabetes information or support?

◯ Yes
◯ No

18. Which social network do you use most often for diabetes information or support? CHECK UP TO 3 CHOICES.

☐ Beyond Type 1
☐ Children with Diabetes
☐ Diabetic Connect
☐ Facebook
☐ Flickr
☐ Glu
☐ Google+
☐ Instagram
☐ LinkedIn
☐ Pinterest
☐ Reddit r/Diabetes
☐ Tu Diabetes
☐ TypeOneNation
☐ Tumblr
☐ Twitter
☐ None
☐ Other (Please specify):

Other diabetes network: __________________________________

19. Do you currently own a smartphone (e.g., iPhone, Android)?

◯ Yes
◯ No

20. With whom are you currently living? Check all that apply.

☐ Spouse (married)
☐ Significant other (unmarried)
☐ Parent(s)
☐ Grandparent(s)
☐ Sibling(s)
☐ Your child/children
☐ Roommate(s) (not related to you)
☐ Alone
☐ Other

Other living with: __________________________________

21. Are you currently pregnant?	◯ Yes<br>◯ No<br>◯ Don't know<br>◯ Not applicable
22. What is the highest level of education you have completed?	◯ Grade school (through grade 8)<br>◯ High school (through grade 12) or equivalent<br>◯ Some college or university<br>◯ Some vocational or trade school<br>◯ Vocational or trade school degree<br>◯ Prefer not to answer
23. What is your current primary occupational status? Check all that apply:	☐ Paid employment (full-time)<br>☐ Paid employment (part-time)<br>☐ Self-employed<br>☐ Full-time homemaker<br>☐ Student<br>☐ Unemployed<br>☐ Other<br>☐ Prefer not to answer
Other occupational status	______________________________
24. What type of drug insurance do you currently have? Select all that apply.	◯ OHIP<br>◯ Private insurance through my employer<br>◯ Private insurance through my parent/guardian<br>◯ Private insurance through my school<br>◯ Private insurance that I purchase directly<br>◯ Interim Federal Health Program<br>◯ Non-Insured Health Benefit (NIHB)<br>◯ Don't know<br>◯ Other<br>◯ None
Other drug insurance	______________________________
25. What is your gender?	◯ Male<br>◯ Female<br>◯ Other(Please specify)<br>◯ Prefer not to answer
Other:	______________________________

26. People living in Canada come from many different cultural and racial backgrounds. Are you:

- ◯ White
- ◯ Chinese
- ◯ South Asian (eg. East Indian, Pakistani, Sri Lankan)
- ◯ Black
- ◯ Filipino
- ◯ Latin American
- ◯ Canadian First Nation (eg. North American Indian, Metis, Inuit)
- ◯ Southeast Asian (eg. Cambodian, Indonesian, Laotian, Vietnamese)
- ◯ Arab
- ◯ West Asian (eg. Afgan, Iranian)
- ◯ Japanese
- ◯ Korean
- ◯ Other (please specify):
- ◯ Prefer not to answer

Other ethnicity

____

12 Month Survey Bridging the Gap to Optimize Care and Outcomes for Youth with Diabetes between Pediatric and Adult Diabetes Care

We are conducting a research study to find out about gaps in services and other issues that young adults with type 1 diabetes face during their transition to adult care. We hope to learn how we can give the best possible care for young adults with type 1 diabetes as they move to adult care.

We are asking you to complete a survey that asks questions about your experiences living with type 1 diabetes. We have asked you to complete the survey once at your last visit to your pediatric clinic and now, 1 year later.

This survey will ask about your current diabetes self-care and support and your healthcare transition. There are 35 questions in this survey and it will take about 20 minutes to complete. By completing the survey you consent to participation as outlined in the particpant letter your received.

You will receive a $10.00 gift card for completing the baseline survey, $10.00 gift card if you complete the 12 month follow-up survey or $25.00 gift card if you complete both the baseline and 12 month follow-up survey.

Please complete the survey below.

Thank you!

Study ID

____
(you received this from the study team)

Today's Date

____

SECTION 1: YOUR CURRENT DIABETES CARE

1. How old are you today

- ◯ 16
- ◯ 17
- ◯ 18
- ◯ 19
- ◯ 20
- ◯ 21
- ◯ 22

To what extent do you feel that you were prepared for the transition to adult diabetes care:
Select one:

- ◯ completely unprepared
- ◯ mostly unprepared
- ◯ neutral
- ◯ mostly prepared
- ◯ completely prepared

2. Have you continued, stopped or started to use any of the following diabetes technologies in the past 12 months:

	never used	continue to use	started to use	stopped using
Insulin pump	◯	◯	◯	◯
Continuous glucose monitoring system (eg Dexcom)	◯	◯	◯	◯
Flash glucose monitoring system (eg Freestyle Libre)	◯	◯	◯	◯

If you have started or stopped using an insulin pump in the last 12 months why? ________________________________

If you have started or stopped using a continuous glucose monitoring system in the last 12 months why? ________________________________

If you have started or stopped using a flash glucose monitoring system in the last 12 months why? ________________________________

3. After your last pediatric diabetes care appointment, about how much time went by before you had your first adult diabetes doctor appointment?

- ◯ Less than 4 months
- ◯ 4-6 months
- ◯ 7-9 months
- ◯ 10-12 months
- ◯ I have not yet seen an adult diabetes doctor
- ◯ not sure

4. If you know the MONTH that you first saw an adult diabetes doctor, please enter it here

- ◯ January
- ◯ February
- ◯ March
- ◯ April
- ◯ May
- ◯ June
- ◯ July
- ◯ August
- ◯ September
- ◯ October
- ◯ November
- ◯ December

5. If you know the YEAR that you first saw an adult diabetes docter, please enter it here:

- ◯ 2018
- ◯ 2019
- ◯ 2020
- ◯ 2021
- ◯ 2022
- ◯ 2023
- ◯ 2024

6. Which of the following best describes your current diabetes doctor?

- ◯ Endocrinologist
- ◯ Family doctor
- ◯ Other (please specify)

Other type of current doctor: __________________________________

7. What is the name of your current diabetes clinic where your saw your diabetes doctor? __________________________________

8. After your last pediatric diabetes care appointment, about how much time went by before you had your first adult diabetes educator visit (eg nurse or dietician)?

- ◯ Less than 4 months
- ◯ 4-6 months
- ◯ 7-9 months
- ◯ 10-12 months
- ◯ I have not yet seen an adult diabetes educator
- ◯ not sure

9. If you know the MONTH that you first saw an adult diabetes educator (eg nurse or dietician) enter it here:

- ◯ January
- ◯ February
- ◯ March
- ◯ April
- ◯ May
- ◯ June
- ◯ July
- ◯ August
- ◯ September
- ◯ October
- ◯ November
- ◯ December

10. If you know the YEAR that you first saw an adult diabetes educator (nurse or dietician) enter it here:

- ◯ 2018
- ◯ 2019
- ◯ 2020
- ◯ 2021
- ◯ 2022
- ◯ 2023
- ◯ 2024

11. What is the name of the clinic where you saw your diabetes educator? __________________________________

12. During the time between your last pediatric diabetes appointment and your first adult diabetes appointment, who prescribed your insulin and diabetes supplies for you? Check all that apply.

- ◯ Pediatric health care provider
- ◯ Adult health care provider
- ◯ Family practice/GP doctor or nurse practitioner
- ◯ Urgent care provider or walk-in clinic
- ◯ Emergency room provider
- ◯ I had enough refills and did not require a new prescription
- ◯ Other (please specify):
- ◯ Don't know

Other provider __________________________________

13. The following is a list of problems that other individuals living with diabetes have said made it hard for them to get established with an adult diabetes provider after leaving their pediatric diabetes provider. How much was each of the following a problem for you?

	Not at all a problem	Small problem	Moderate problem	Major problem
a. I didn't have a name for a new adult provider	◯	◯	◯	◯
b. I didn't know how to contact the new adult provider	◯	◯	◯	◯
c. I couldn't get an appointment with the new adult provider	◯	◯	◯	◯
d. I forgot to make an appointment or had other priorities	◯	◯	◯	◯
e. I felt upset about leaving my pediatric diabetes providers	◯	◯	◯	◯
f. Other problems for you (please specify):	◯	◯	◯	◯

Other: ______________________________

14. Overall, how satisfied or dissatisfied were you with how your transition to adult diabetes care went?

- ◯ Completely dissatisfied
- ◯ Mostly dissatisfied
- ◯ Neutral
- ◯ Mostly satisfied
- ◯ Completely satisfied

15. Approximately what was your hemoglobin A1c the last (most recent) time it was checked?

- ◯ Less than 7.0%
- ◯ 7.0-7.9%
- ◯ 8.0-8.9%
- ◯ 9.0-9.9%
- ◯ 10.0-10.9%
- ◯ 11.0% or more
- ◯ Do not know

16. To what extent are your parents/guardians currently involved in your diabetes care?

- ◯ Not at all involved
- ◯ Somewhat involved
- ◯ Moderately involved
- ◯ Very involved

17. How involved would you like your parents/guardians to be in your diabetes care?

- ◯ Not at all involved
- ◯ Somewhat involved
- ◯ Moderately involved
- ◯ Very involved

18. In general, how often do you feel stressed because of your diabetes?

- ◯ Never
- ◯ Rarely
- ◯ Sometimes
- ◯ Often
- ◯ Always

19. How often does our diabetes prevent you from doing the following (select one):

	< once per month	1-2 times per month	3-4 times per month	> 4 times per month
Exercise	○	○	○	○
Sleep	○	○	○	○
Attend school	○	○	○	○
Attend work	○	○	○	○
Participate in social activities	○	○	○	○

20. In the last year, have you experienced a major change in your life situation that caused you to feel "stressed" or have a physical, mental, or emotional response for an extended period of time?

- ○ Yes
- ○ No
- ○ Don't know

21. If YES: In the past year, which of the following events have you experienced? Please check all that apply.

- ☐ Serious illness or injury in me
- ☐ Serious illness or injury in a family member
- ☐ Hospitalization of a family member
- ☐ Death of a family member
- ☐ Death of a friend
- ☐ Separation or divorce of parents
- ☐ Split up with boyfriend/girlfriend
- ☐ Moved to a new home
- ☐ Started attending a new school
- ☐ Started working at a new job
- ☐ Problems at work or school
- ☐ Lost my job
- ☐ Family member lost his/her job
- ☐ Financial problems in the family
- ☐ Legal problems in the family
- ☐ Other event (please specify):

Other event: ______________________________

22. Do you currently use any general online social networks (e.g., social networks like Facebook that do not focus on diabetes)?

- ☐ Yes
- ☐ No

23. Which general social networks do you use most often? CHECK UP TO 3 CHOICES.

- ☐ Facebook
- ☐ Flickr
- ☐ Google+
- ☐ Instagram
- ☐ LinkedIn
- ☐ Pinterest
- ☐ Snapchat
- ☐ Tinder
- ☐ Tumblr
- ☐ Twitter
- ☐ None
- ☐ Other (please specify):

Other general network: ______________________________

24. Do you currently use any social networking sites for diabetes information or support?

- ○ Yes
- ○ No

25. Which social network do you use most often for diabetes information or support? CHECK UP TO 3 CHOICES.

- ☐ Beyond Type 1
- ☐ Children with Diabetes
- ☐ Diabetic Connect
- ☐ Facebook
- ☐ Flickr
- ☐ Glu
- ☐ Google+
- ☐ Instagram
- ☐ LinkedIn
- ☐ Pinterest
- ☐ Reddit r/Diabetes
- ☐ Tu Diabetes
- ☐ TypeOneNation
- ☐ Tumblr
- ☐ Twitter
- ☐ None
- ☐ Other (Please specify):

Other network: ______________________________

26. Do you currently own a smartphone (e.g., iPhone, Android)?

- ☐ Yes
- ☐ No

SECTION 5: DEMOGRAPHICS

27. With whom are you currently living? Check all that apply.

- ☐ Spouse (married)
- ☐ Significant other (unmarried)
- ☐ Parent(s)
- ☐ Grandparent(s)
- ☐ Sibling(s)
- ☐ Your child/children
- ☐ Roommate(s) (not related to you)
- ☐ Alone
- ☐ Other (please specify):

Other living arrangement: ______________________________

28. Are you currently pregnant?

- ◯ Yes
- ◯ No
- ◯ Don't know
- ◯ Not applicable

29. What is the highest level of education you have completed?

- ◯ Grade school (through grade 8)
- ◯ High school (through grade 12) or equivalent
- ◯ Some college or university
- ◯ Some vocational or trade school
- ◯ Vocational or trade school degree
- ◯ College or University
- ◯ Prefer not to answer

30. What is your current primary occupational status? Check all that apply.	◯ Paid employment (full-time)<br>◯ Paid employment (part-time)<br>◯ Self-employed<br>◯ Full-time homemaker<br>◯ Student<br>◯ Unemployed<br>◯ Other (please specify):<br>◯ Prefer not to answer
Other occupational status:	__________________________________
31. What type of drug insurance do you currently have? Select all that apply.	◯ OHIP<br>◯ No insurance<br>◯ Private insurance through my employer<br>◯ Private insurance through my parent/guardian<br>◯ Private insurance through my school<br>◯ Private insurance that I purchase directly<br>◯ Interim Federal Health Program<br>◯ Non-Insured Health Benefit (NIHB)<br>◯ Don't know<br>◯ Other
Other drug insurance:	__________________________________
32. What is your gender?	◯ Male<br>◯ Female<br>◯ Other (please specify):<br>◯ Prefer not to answer
Other:	__________________________________
35. People living in Canada come from many different cultural and racial backgrounds. Are you:	◯ White<br>◯ Chinese<br>◯ South Asian (eg. East Indian, Pakistani, Sri Lankan)<br>◯ Black<br>◯ Filipino<br>◯ Latin American<br>◯ Canadian First Nation (eg. North American Indian, Metis, Inuit)<br>◯ Southeast Asian (eg. Cambodian, Indonesian, Laotian, Vietnamese)<br>◯ Arab<br>◯ West Asian (eg. Afgan, Iranian)<br>◯ Japanese<br>◯ Korean<br>◯ Other (please specify):<br>◯ Prefer not to answer
Other ethnicity:	__________________________________
