# Supplementary material for: Testing an audit and feedback-based intervention to improve glycemic control after transfer to adult diabetes care: protocol for a quasi-experimental pre-post design with a control group
Source: BMC Health Serv Res. 2019 Nov 25;19:885. doi: 10.1186/s12913-019-4690-0 (PMC6878686; doi:10.1186/s12913-019-4690-0)
Supplement: Supplementary file 3 — Additional file 3. Mental Health Diagnostic Codes. [file 12913_2019_4690_MOESM3_ESM.docx]

Additional file 3: Mental Health Diagnostic Codes

**Psychotic Disorders**

295 Schizophrenia

296 Manic-depressive psychoses, involutional melancholia

297 Other paranoid states

298 Other psychoses

**Mood and Anxiety**

300 Anxiety neurosis, hysteria, neurasthenia, obsessive-compulsive neurosis, reactive depression

309 Adjustment reaction

311 Depressive disorder

**Behavioral Disorders**

313 Behaviour disorders of childhood and adolescence

314 Hyperkinetic syndrome of childhood

**Substance Use Disorders**

291 Alcoholic psychosis, delirium tremens, Korsakov's psychosis

292 Drug psychosis

303 Alcoholism

304 Drug dependence

305 Tobacco abuse

**Social Problems**

897 Economic problems

898 Marital difficulties

899 Parent-child problems

900 Problems with aged parents or in-laws

901 Family disruption/divorce

904 Social maladjustment

905 Occupational problems

906 Legal problems

909 Other problems of social adjustment

**Other**

301 Personality disorders

302 Sexual deviations

306 Psychosomatic illness

307 Habit spasms, tics, stuttering, tension headaches, anorexia nervosa, sleep disorders, enuresis

902 Education problems
